# CyberKnife multisession stereotactic radiosurgery and hypofractionated stereotactic radiotherapy for perioptic meningiomas: intermediate-term results and radiobiological considerations

**DOI:** 10.1186/s40064-015-0804-2

**Published:** 2015-01-30

**Authors:** Alfredo Conti, Antonio Pontoriero, Federica Midili, Giuseppe Iatì, Carmelo Siragusa, Chiara Tomasello, Domenico La Torre, Salvatore M Cardali, Stefano Pergolizzi, Costantino De Renzis

**Affiliations:** Department of Neurosurgery, University of Messina, AOU “Policlinico G. Martino”, Via Consolare Valeria 1, 98125 Messina, Italy; Department of Radiation Oncology, University of Messina, AOU “Policlinico G. Martino”, Via Consolare Valeria 1, 98125 Messina, Italy; Department of Medical Physics, University of Messina, AOU “Policlinico G. Martino”, Via Consolare Valeria 1, 98125 Messina, Italy; Department of Oncology, University of Messina, AOU “Policlinico G. Martino”, Via Consolare Valeria 1, 98125 Messina, Italy

**Keywords:** Meningioma, CyberKnife, Radiation induced optic neuropathy, Hypofractionated stereotactic radiotherapy, Radiobiology

## Abstract

Single fraction radiosurgery is conventionally precluded for lesions lying <2-3 mm of the anterior visual pathway because of the radiosensitivity of the optic nerve. We analyzed a series of 64 patients with “perioptic” meningiomas treated by CyberKnife multisession radiosurgery and hypofractionated stereotactic radiotherapy (hSRT). Between July 2007-May 2010, patients were treated using conventional multisession Cyberknife schemes (2–5 fractions) and results were retrospectively analyzed. A radiobiological model was then developed to estimate the best tumor control probability (TCP)/ normal tissue complication probability (NTCP) for these lesions. Resulting dose/fraction schemes were applied to patients treated between May 2010 and July 2014. Data were prospectively collected

Twenty-five patients were included in the retrospective part of the study. Median tumor volume was 4.95 cc; median dose was 23.0 Gy and median number of fraction was 5 (range 2–5). No patient had visual deterioration at mean follow-up of 60 ± 12 months. Tumor control was achieved in all cases. Thirty-nine patients were treated according the radiobiology model and results prospectively analyzed. Median tumor volume was 7.5 cc, median dose 25.0 Gy and mean number of fraction 5 (range 3–15). No patient had visual deterioration or tumor progression at mean follow-up of 17 ± 10 months.

Conventional multisession CyberKnife treatments (2–5 fractions) provided satisfactory results. Nonetheless, our estimation of TCP suggests the use of higher doses to grant long-term disease control. To achieve higher equivalent doses without significantly increasing the NTCP, we suggest the use of a greater number of fractions, moving to hSRT, in tumors in which the encasement of optic nerves is presumed.

**Electronic supplementary material:**

The online version of this article (doi:10.1186/s40064-015-0804-2) contains supplementary material, which is available to authorized users.

## Background

Stereotactic radiosurgery has progressively emerged as both an adjuvant treatment modality for residual tumors and an effective primary treatment of properly selected meningiomas. Because of the sensitivity to radiation of the optic apparatus, radiosurgery is conventionally precluded for “perioptic” meningiomas, namely for lesions lying <3 mm of the anterior visual pathways (Leber et al. 1995; Stafford et al. 2003). In fact, doses higher than 10.0-12.0 Gy in a single fraction carry a significant risk of visual injury (Leber et al. 1995; Stafford et al. 2003; Tishler et al. 1993; Pollock et al. 2008). The dose gradient that can be achieved with single-session photon radiosurgery is typically inadequate to grant the delivery of sufficient dose of radiations to the tumor while maintaining harmless doses to the optic nerve.

Perioptic meningiomas that are not amenable to surgical treatment have been, therefore, treated with fractionated external beam radiation therapy (EBRT). However, with conventional EBRT tumor control may not be quite as good as with radiosurgery and complications are possible (Minniti et al. 2009, 2011). Goldsmith et al. (1994) identified complications in five out of 140 patients (3.6%) attributable to EBRT. Authors described retinopathy in 2 patients, optic neuropathy in one, and cerebral necrosis in 2 patients (Goldsmith et al. 1994). Patients with parasellar meningiomas are also at risk of late hypopituitarism and should be carefully assessed long-life after radiotherapy. Neurocognitive dysfunction is a recognized consequence of large volume radiotherapy for brain tumors (Crossen et al. 1994) and has been occasionally reported in irradiated patients with meningiomas, especially impairment of short-term memory (Dufour et al. 2001; Maguire et al. 1999; Maire et al. 1995). High dose radiation may be associated with the development of a second brain tumor. In a large series of 426 patients with pituitary adenomas who received conventional RT at the Royal Marsden Hospital between 1962 and 1994, Minniti et al. reported that the risk of second brain tumors was 2.0% at 10 yr and 2.4% at 20 yr, measured from the date of RT (Minniti et al. 2005).

The emergence of image-guided and frameless technology enabled the principles of multisession stereotactic treatments, conventionally consisting of 2–5 fractions of 4.0-10.0 Gy each, whose goal is a highly conformal irradiation, actually comparable to that of single fraction radiosurgery, with a putative lower toxicity on the optic apparatus (Conti et al. 2009).

Recently, short to midterm follow up data on multisession radiosurgery of perioptic meningiomas have been reported (Romanelli et al. 2007; Colombo et al. 2009; Adler et al. 2006). Results are encouraging, but a number of issues remain to be definitively addressed. In particular, the radiobiological bases and the dose/fraction schemes to be used in a hypofractionated treatment of meningiomas still need to be clarified (Shrieve et al. 2004; Conti et al. 2012, 2013; Alafaci et al. 2014).

We retrospectively analyzed a series of patients affected by perioptic meningiomas who were treated using multisession radiosurgery using a robotic, frameless device (CyberKnife, Accuray Inc., Sunnyvale, CA) in the first four years of activity. We then developed a radiobiological model based on our results and available literature. Treatments based on the radiobiological model were performed in most recent cases that were prospectively analyzed.

## Results

Sixty-four patients with perioptic meningioma were included in the study. All tumors were located within 2 mm of the optic apparatus and included tuberculum sellae, cavernous, spheno-cavernous, spheno-petro-clival, anterior clinoid, optic-nerve sheath meningiomas. Mean tumor volume was 11.5 ± 15 cc.

Twenty-five patients were treated between July 2007-May 2010 and were retrospectively analyzed. Thirty-nine consecutive patients underwent CyberKnife treatment according to a dose/fraction scheme selected on the base of a radiobiological model to achieve a tumor biologically equivalent dose (BED) of 100.0 Gy_2_, while maintaining a normal tissue complication probability (NTCP) <2%.

### Retrospective data (2007–2010)

Twenty-five patients were treated using conventional CyberKnife multisession radiosurgery schemes (2–5 fractions). The median follow-up was 57.5 months (range 48–82 months). The median volume of the treated lesions was of 4.95 cc (range 0.3-18.8 cc); median marginal dose was 23.0 Gy (range 18–34 Gy), with a median prescription isodose line of 75% (range: 63–80%). Mean BED was 82.8 Gy_2_, median BED was 87.5 (range 72.0-102.0 Gy_2_).

Twenty-eight percent (7 of 25 patients) of the patients presented visual acuity or visual field impairment before treatment. Twelve percent (3 of 25 patients) had no serviceable vision monolaterally, 4% bilaterally. Twelve percent had significant limited visual acuity or visual field deficit with still serviceable function mono- or bilaterally. No patients presented clinical and laboratory evidence of hypopituitarism. Three patients (12%) had oculomotor nerves deficits.

In a prescription range of 18.0-25.0 Gy, doses used were: 18.0 Gy in two fractions, 18.0-21.0 Gy in 3 fractions, 20.0-22.0 Gy in 4 fractions and 23.0-25.0 Gy in 5 fractions. The maximal accepted doses to the optic nerve and chiasm were: 10.0 Gy in 2 fractions, 15.0 Gy in 3 fractions, 20.0 Gy in 4 fractions, 25.0 Gy in 5 fractions. None of the patients showed visual field or acuity deterioration, whereas in 20% of patients visual field or acuity significantly improved after radiotherapy treatments. No tumor progressed at a median follow up of 57.5 months. No patient developed new pituitary function deficits at the latest follow up. Two of three patients had partial improvement of the third nerve deficits presented preoperatively. Two patients (8%) complained of transient trigeminal neuralgia lasting 6 to 12 months after treatment. No radiation-induced signal change could be seen on follow up MRI studies.

### Radiobiological data

**Figure 1:**
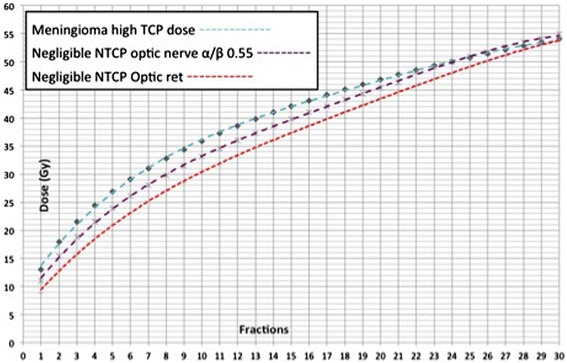
**Graph showing total doses of radiation associated with a predicted biologically equivalent effect on meningioma control for various numbers of equal daily fractions (turquoise line).** Calculations were based on the linear quadratic model considering isoeffective 13–14 Gy delivered in single fraction and 54 Gy in 30 fractions. Each dose corresponds to a biologically equivalent dose BED of 102.6 Gy an α/β of 2. Low risk of optic neuropathy as a function of the number of equal daily fractions were also calculated to understand the range of dose to be delivered to the optic apparatus at a specific dose/fraction scheme. Calculations were based on the “optic ret” model of Goldsmith et al. (1992), with an equivalent optic ret dose of 890 (red line) and using the linear quadratic model and considering isoeffective 11 Gy delivered in single fraction and 50 Gy in 25 fractions (purple line).

We calculated the α/β of meningiomas using two irradiation regimens with equivalent TCP using the equations 5 and 6 from the Additional file (Additional file 1). Recent evidence clearly demonstrate that single fraction SRS treatment regimens ranging between 13–14 Gy grant high TCP (Minniti et al. 2009), that are similar to that of 54.0 Gy in 30 fractions. Using the isoeffect model (eq. 5 and 6) and considering 13.0-14.0 Gy in single fraction equivalent to 54.0 Gy/30 fractions in terms of efficacy, the α/β value for meningiomas ranges 1.75-2.46. Adopting an α/β of 2, the target BED to which aim to when adopting different fractionation schemes is 102.6 Gy (Figure 1). The regimen used in our series until June 2011 had an average BED of 84.7 Gy using this α/β of 2. In particular the BED of 25.0 Gy in 5 fractions is 87.5 Gy_2_.

**Figure 2:**
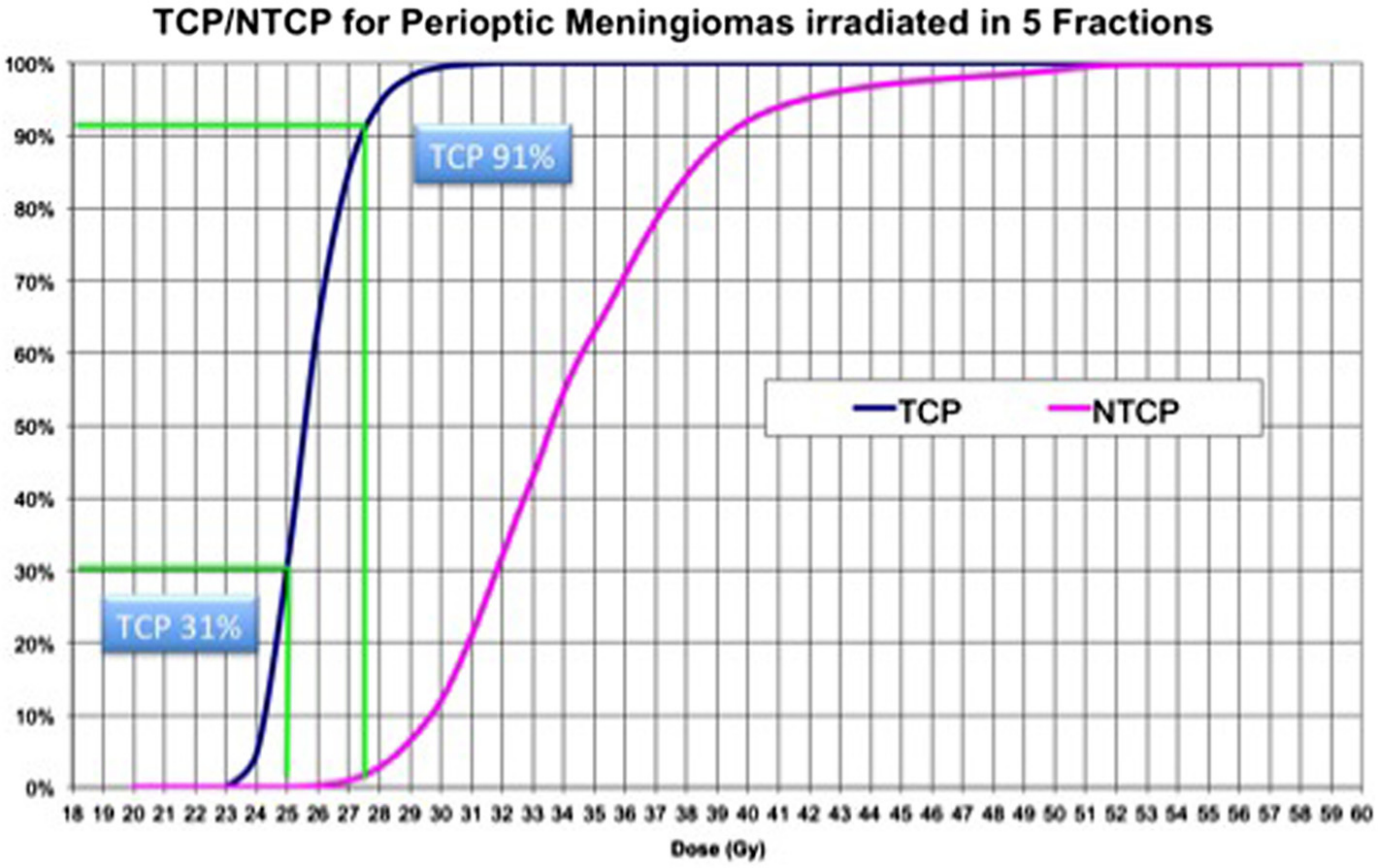
**Tumor control probability (TCP) and normal tissue complication probability (NTCP) curves for meningioma treated using a 5 fractions scheme.** The TCP curve was built using an α/β of 2 for meningiomas. TCP values are 4.8% for 2400 cGy, 31% for 2500 cGy, 91% for 2750 cGy and 100% for 3300 cGy. The NTCP of the optic nerve and chiasm using the same irradiation scheme, according to the Lyman-Kutcher-Burman model, results respectively 0.02% for a dose of 2400 Gy, 0.12% for 2500 cGy, 2.98% for 2750 cGy and 43% for 3300 cGy.

We calculated the TCP of this irradiation scheme according to the eq.15. The delivery of 25.0 Gy in 5 fractions has a TCP of 31% (Figure 2). The addition of 0.5 Gy per fraction, with a total dose of 27.5 Gy, allows an increase of the TCP to 91%.

We used the linear quadratic model (LQ) to calculate the tolerance of the optic apparatus. The isoeffect model was used to estimate the α/β of the optic nerve and chiasm and the threshold maximal doses (Dmax) using the equations 5 and 6, similarly to what we did for the tumor. We considered two irradiations regimes that are similar in terms of complication rate. Considering isoeffective, in terms of NTCP, a Dmax of 11.0 Gy in single fraction (Stafford et al. 2003) and 50.0 Gy in 25 fractions (5% complication probability at 5 years (Emami et al. 1991)). Accordingly, the α/β of the optic nerve and chiasm turned out to be 0.55 Gy. Using this α/β, the isoeffective BED is and the Dmax values to be used in hypofractionated treatments, having a NTCP similar to that of 50.0 Gy in 25 fractions are: 15.5 Gy in 2 fractions, 19.0 Gy in 3 fractions, 22.0 Gy in 4 fractions, 24.0 Gy in 5 fractions (Figure 1).

We calculated the NTCP of those doses on the optic apparatus according to the Lyman-Kutcher-Burman model (Kutcher and Burman 1989; Burman et al. 1991; Lyman 1985). Using the parameters provided in the Emami study (Emami et al. 1991), a Dmax of 24.0 Gy in 5 fractions to the optic nerve and chiasm resulted in a NTCP of 0.02%, that is 0.12 for 25.0 Gy in 5 fractions and 2.98% for 27.5 Gy in 5 fractions (Figure 2).

Using 15 fractions and delivering 40.0 Gy (BED = 93 Gy_2_) to the tumor and encased nerves, we calculated a TCP of 64% and NTCP of 1.8%.

### Prospective data (2010–2014)

Thirty-nine consecutive patients were included in this study. The median follow-up was 15 months (mean 17 ± 10; range 3–38 months). The median volume of the treated lesions was of 7.5 cc (range 1.2-44.1 cc). Median marginal dose was 25.0 Gy (range 19.5-40.0 Gy) with a median number of fraction of 5 (range 3–15) and a prescription isodose line of 75% (range: 62–82%). In a range of 18.0-40.0 Gy, doses delivered were: 18.0 Gy in two fractions, 18.0-21.0 Gy in 3 fractions, 20.0-22.0 Gy in 4 fractions, 25.0 Gy in 5 fractions, 27.5 Gy in 6 fractions, 30.0 Gy in 9 fractions, 34.0 Gy in 10 fractions, 40.0 Gy in 15 fractions. Mean BED was 91.3, median BED was 87.5 Gy_2_ (Range 60.0-120.0 Gy_2_).

Twenty-three percent (9 of 39 patients) of the patients presented visual acuity or field impairment. Eigth percent (3 of 39 patients) had no serviceable vision monolaterally, 5% (2 patients) bilaterally. Ten percent (4 patients) had significant limited visual acuity or visual field deficit with still serviceable sight mono or bilaterally.

**Figure 3:**
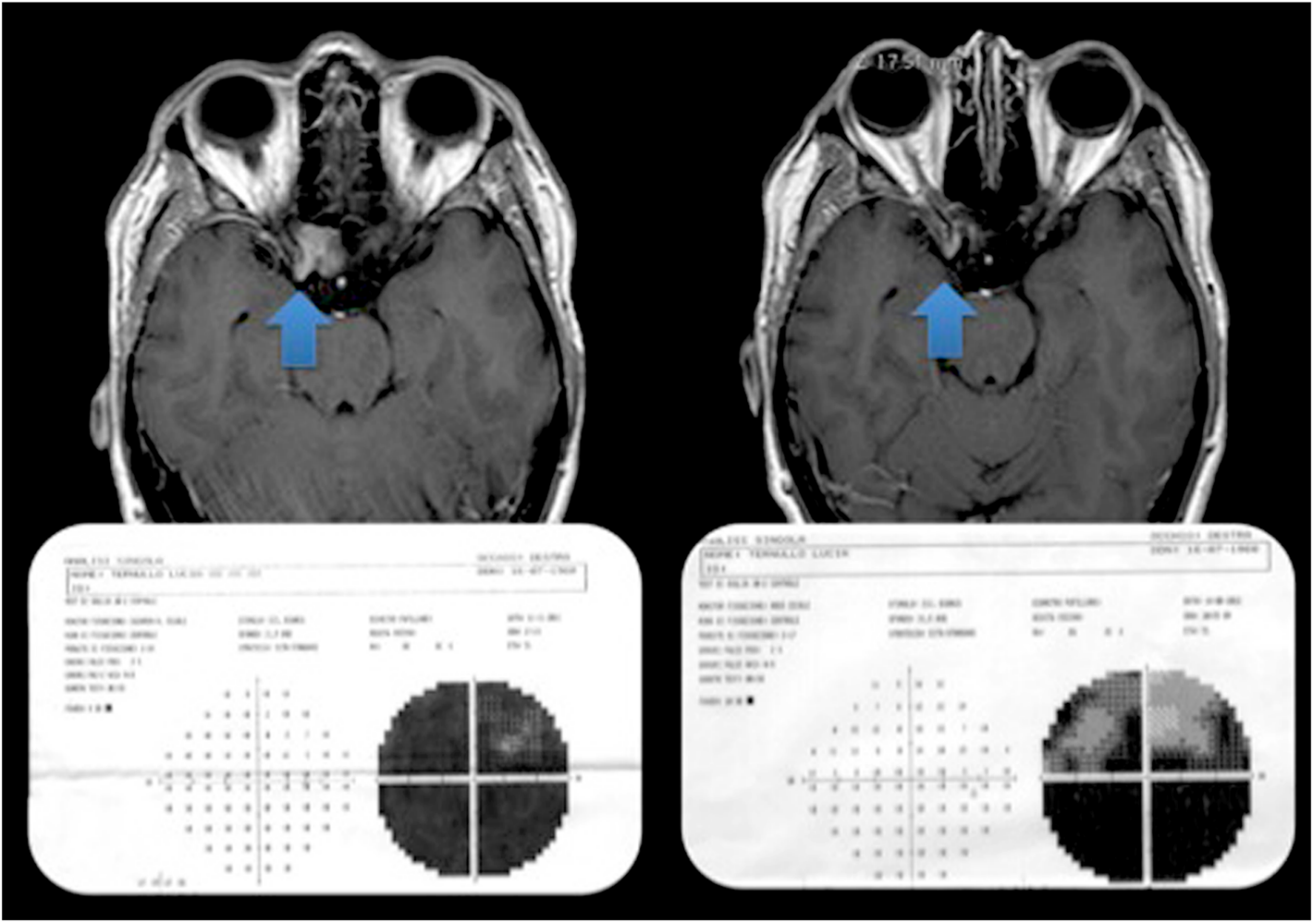
**Left.** A meningioma invading the right optic canal with optic nerve encasement causing a significant visual impairment. The patient underwent hypofractionated CyberKnife treatment with the delivery of 40 Gy in 15 fractions Right. Eight months after the treatment, the shrinkage of the tumor was recorded together with a partial recovery of the visual field.

The maximal admitted doses to the optic nerve and chiasm were: 10.0 Gy in 2 fractions, 15.0 Gy in 3 fractions, 20.0 Gy in 4 fractions, 25.0 Gy in 5 fractions, 30.0 Gy in 9 fractions, 34.0 Gy in 10 fractions and 40.0 in 15 fractions. To date, no patient showed visual field or acuity deterioration. Visual improvement (visual acuity and/or field) was recorded in 18% of patients. No tumor progression was recorded, whereas tumor shrinkage was obtained in 28% (Figure 3). No patient developed new pituitary function deficits at the latest follow up. Two of three patients had partial improvement of the oculomotor nerve deficits presented preoperatively. Three patients (7.7%) complained of transient trigeminal neuralgia lasting 6 to 12 months after treatment controlled with low doses (200–400 mg) of carbamazepine. No radiation-induced signal change could be seen on follow up MRI studies.

## Discussion

Multisession radiosurgery, 2–5 fractions as those typically delivered with the CyberKnife, controlled tumor growth without optic nerve toxicity in a group of 25 patients with a median follow up of 57.5 months (range 48–82). According to our theoretical model, the late tumor control could be achieved by dose/fraction scheme providing a BED >100 Gy_2_, i.e. 27.5 Gy in 5 fractions. Contextually, the dose to the optic nerves should be maintained to <5 Gy/f to achieve a marginal risk radio-induced optic neuropathy. Accordingly, the safest treatment should provide a dose gradient to have a decrease of 10% of the dose between the tumor and the nerves. Whenever this dose gradient cannot be achieved, i.e. when nerves cannot be clearly distinguished because with respect of the tumor, we increased the number of fractions, namely moving to hSRT, to reduce the NTCP while maintaining a high TCP. This resulted in satisfactory results in the mid-term without tumor progression and with 18% of patients in whom vision has improved.

The first series reporting of the use of CyberKnife multisession radiosurgery of perioptic tumors was that of the Stanford University, were the CyberKnife radiosurgery system was developed (Adler et al. 2006). Adler et al. treated 49 patients with perioptic tumors, including 27 meningiomas, in 2–5 sessions with a cumulative average marginal dose of 20.3 Gy. At an intermediate-term follow-up of 49 months (range, 6–96 months), vision was unchanged post-radiosurgery in 38 patients, improved in eight (16%), and worse in three (6%). In two cases, the deterioration was attributed to tumor progression. In this series, the use of a more aggressive protocol (21.0 Gy delivered in 3 sessions) was associated with loss of vision in one patient. In contrast, the protocol used by Romanelli et al. in four patients with optic nerve sheath meningiomas and consisting of 20.0 Gy delivered in 4 sessions induced fast resolution of visual symptoms and good preservation of vision after a mean follow-up duration of 37 months (Romanelli et al. 2007). In the largest series on meningiomas treated with the CyberKnife, Colombo et al. treated 29 perioptic meningiomas using up to 5 fractions and up to 25.0 Gy (Colombo et al. 2009). Authors recorded a visual worsening in 2 cases (1% of the overall series), again this was ascribed to the tumor progression. Marchetti et al. treated 21 patients with optic nerve sheath meningiomas with 25.0 Gy in 5 fractions (Marchetti et al. 2011). No patient had visual deterioration at a mean follow up of 30 months, visual improvement was recorded in 35% of patients.

We treated 64 patients with perioptic meningiomas in 2–15 fractions. Neither radio-induced optic neuropathy, nor tumor progression was recorded at an overall mean follow up of 32 ± 23 months.

Other perioptic tumors have been treated using similar protocols. Killory et al. treated 20 patients with pituitary adenomas located within 3 mm from the optic chiasm with the CyberKnife (Killory et al. 2009). In all cases Authors used 25.0 Gy in 5 fractions under the hypothesis that the optic apparatus may tolerate 5 consecutive daily fractions of 5.0 Gy. At a median follow up of 26 months no patient’s vision deteriorated.

Despite the limited number of patients with sufficient follow up, some considerations can be done based on the above-mentioned literature data. The use of up to 5.0 Gy per fraction in a 5-session treatment is commonly used, and it is felt safe for the optic nerve. Fundamentally, this is confirmed by clinical data. Considering only mid-term data, no patient deteriorated with 22.0-25.0 Gy in 5 fractions, namely with a BED ranging 70.0-87.5 Gy_2_. One patient deteriorated with 21.0 Gy in 3 fractions and a corresponding BED of 94.5 Gy_2_. This is in line with our estimate of the optic nerve tolerance in hypofractionated treatment, which is based on the use of the isoeffective treatments and the LQ model.

More importantly, two patients in the series of Adler et al. and two patients in the series of Colombo et al. had tumor progression-related visual deterioration. Accordingly, the issue of tumor progression seems to be more relevant than the radio-induced optic neuropathy at the currently used dose/fraction schemes. Also this point is in line with our evaluation of the TCP. In fact, according to our estimate, the BED used in the available literature, range 70.0-87.5 Gy_2_ and have a TCP ≤ 31%. This point is particularly relevant since the lack of control of tumor progression in perioptic meningiomas is not inconsequential, and was the primary cause of functional deterioration in previous reports (Adler et al. 2006; Colombo et al. 2009).

The supplement of 0.5 Gy/fraction in a 5-fraction regimen, with a total dose of 27.5 Gy, will raise the TCP to 91%, according to our model (Figure 2). However, this same dose cannot be delivered to the optic nerve since its BED is 103 Gy_2_. The delivery of a similar dose to the optic nerve carries a risk of neuropathy according to our NTCP model (Figure 2).

We calculated that 24 Gy in 5 fractions carry a negligible risk of toxicity (Figure 2). Using the NTCP model, the risk associated to the delivery of 27.5 Gy is about 100 times higher. Accordingly, the dose gradient that should be achieved is of 10-15% of the marginal dose. Using an inverse planning algorithm it is possible to achieve this gradient, provided that a minimal distance between the tumor and nerves remains. When the nerves are encased by the tumor, 5 fractions could not be sufficient to grant the best possible TCP/NTCP ratio. We escalated the dose up to 40.0 Gy in 15 fractions according to the model with satisfactory mid-term clinical results (Figure 3).

Our study has different limitations. Firstly, meningioma is a tumor with low-proliferative index, low α/β ratio, and is late responding tissue. In this setting, the largest possible session dose has a radiobiology advantage. Furthermore, long-term results are necessary to demonstrate the real efficacy profile of hypofractionated regimens on such tumors. Nevertheless, we should consider that perioptic meningiomas are not susceptible of single fraction radiosurgery and that we advocate the use of multisession radiosurgery and hSRT to reduce the risk of toxicity to normal tissue.

Secondly, the model was obtained creating curves based on the interpolation of clinical data of patients who underwent single fraction SRS and conventional EBRT treatments. Clinical results obtained directly in the realm of hypofractionation may change, however, our understanding of radiobiology of meningiomas and optic nerves.

We used radiobiological models developed for homogeneous dose distributions, but doses used with radiosurgery are typically inhomogeneous. We used the prescription isodose (i.e. 80% isodose) to estimate the TCP, but doing so we possibly overestimated the TCP, because of the presence of tumor-lets receiving doses lower than that prescribed. In addition, we used the maximal dose delivered to the optic nerve to estimate the NTCP, but the dependency of toxicity on the volume of the irradiated nerve remains an important issue. Actually, many of the studies on which we based evaluation of the tolerance were performed in an era before the routine use of CT or MRI-based planning, dose-volume histogram analysis, and steep dose gradients across structures. Optic nerves have a “parallel architecture”, but it was considered as a “serial” organ, with no volume effect according to the Emami study (Emami et al. 1991). Nevertheless, treatments with rapid dose gradients limiting volume irradiation may results in limited toxicity or incomplete visual field defects, which in some circumstances, can be very modest or considered clinically acceptable. Although the model was not designed taking into account doses to the pituitary gland and no patient developed hypopituitarism, this is a potential complication of the treatment at the doses we selected. We advocate to limit the irradiation of the sella and pituitary stalk whenever possible and the long-term assessment of pituitary function.

## Conclusions

We treated a substantial number of patients with perioptic meningiomas. A subgroup of 25 patients with a median follow up of 57.5 months (range 48–82) showed 100% progression free survival without toxicity with a median dose of 23.0 Gy delivered in 4.5 fractions. Considering that tumor progression could cause irreversible functional deterioration, we suggest providing a BED >100 Gy_2_, i.e. 27.5 Gy in 5 fractions, but the dose to the optic nerves should be maintained to <5.0 Gy/f to avoid optic neuropathy. Accordingly, a dose gradient of at least 10% of the marginal tumor dose is needed. In tumors in which an encasement of the optic nerves is supposed, based on neuroimaging, a higher number of fractions is necessary. In the subgroup of patients prospectively collected and treated according to the model no visual impairment was recorded. Longer follow up are, however, necessary to estimate the probability of tumor control.

## Material and methods

### Patients

Between July 2007 and July 2014, 64 patients with “perioptic” meningiomas were treated by multisession radiosurgery (2–5 fractions) or hypofractionated stereotactic radiotherapy (hSRT) using a CyberKnife system. Clinical, demographic characteristics of patients and treatment parameters are summarized in Table 1. From 2007 to May 2010, patients were treated with conventional CyberKnife multisession schemes (2–5 fractions) and were retrospectively analyzed. From June 2010 to July 2014, the dose/fraction scheme was selected according the results of a radiobiological model described in the Additional file (Additional file 1). The study has been approved by the Local Ethics Committee (Comitato Etico Interaziendale della Provincia di Messina; http://www.polime.it/comitato_etico_interaziendale). Patients signed an informed consent form.

**Table 1:** **Summary of demographic characteristics and treatment parameters of 64 patients**

	**Male**	**29**
	**Female**	**35**
**Age** (yr)	Median	62
	Range	23-84
**Pre-treatment**	Surgery	36
	Radiotherapy	7
**Target**	Median Volume	7.4 cc (range 0.3-44.4 cc)
	Median Marginal Dose	25Gy (range 18-40Gy)
	Median Isodose line	75% (range 62-82%)
	Median BED	87.5 Gy_2_ (range 72–120)
	**Number of Fractions**	**Number of patients (%)**
	2	1 (1.6)
	3	13 (20.3)
	4	3 (4.7)
	5	40 (62.5)
	9	1 (1.6)
	10	3 (4.7)
	15	3 (4.7)

### Imaging and treatment

We used a Cyberknife system, an image-guided, frameless LINAC-based, 6 MV radiosurgery system that uses inverse planning for the delivery of radiation to a defined target volume. The patient’s head was immobilized with a thermoplastic mask for imaging acquisition and treatment. The neuroimaging technique consisted of a thin-section, contrast-enhanced, multiplanar reconstruction-gradient echo volumetric study conducted on a Siemens Magnetom 1.5-T MR imaging system (Siemens, Erlangen, Germany), performed at the following parameters: TR 9.7 ms, TE 4 ms, matrix 200 × 256, flip angle 1, orientation sagittal. A Multislice contrast-enhanced CT was also performed using a multislice scanner Siemens Sensation 16 (Siemens).

Contouring of the tumor and the critical volumes was performed on the co-registered MR and CT dataset. Manual contouring was done in the axial plane with simultaneous display of contours on reconstructed orthogonal images.

### Selection o doses and fractionations

The selection of the marginal and maximal doses and the number of sessions were influenced by multiple factors including tumor volume, volume of the irradiated optic nerve, visual function, as well as history of previous radiation therapy. After considering those variables, from June 2010 we selected doses of radiation and number of fractions based on a radiobiological model described in the Additional file 1 (supplemental material) and graphically displayed in Figure 1. In brief, we tried to achieve doses of radiations equivalent to 13.0 Gy in single fraction (conventional SRS) or 54.0 Gy in 30 fractions (conventional fractionation). The equivalence was obtained through the radiobiological model.

An inverse planning algorithm using a nonisocentric technique determined the optimal treatment planning program. The ray-tracing algorithm was routinely used to this purpose. Some of the methods used include: 1) selection of the size and number of collimators, balancing the necessity of coverage, reduction of the number of radiation beams and monitor units with the necessity of steep dose gradients in specific areas; 2) the addition of tuning structures to reduce uncontrolled dose diffusion; 3) definition of dose constraints and their weight to the target volume and critical structures; and 4) maximization of resolution of dose calculation using the smallest calculation grid and calculation grid expansion to evaluate distant isodose distribution.

### Patient assessment

All patients underwent serial neurological, endocrinological and ophtalmological examinations. Radiological assessment consisted of a contrast-enhanced MRI scan obtained at 3 months and then every 6 months for 2 years. Afterwards, contrast-enhanced brain MRI scans had been obtained yearly. Follow up MRI studies consisted of contrast-enhanced T1 weighed, T2w, Proton Density and FLAIR sequences in all cases.

Ophtalmological follow up consisted of visual acuity studies and computerized visual field perimetry test serial assessments performed by an opthalmologist. Visual acuity was considered normal if >20/80; visual perimetry was qualitatively assessed.

The first assessment was obtained 9 months after the treatment. Examinations were then obtained at the same time intervals than the MRI, every six months for two years, then yearly. Endocrinological follow up, consisting of the assessment of thyroid hormones, prolactin, cortisol, Dehydroepiandrosterone (DHEA), IGF-1 serum levels, were obtained at the same time intervals as the ophthalmological follow up. Normal values range could be different in different laboratories and were evaluated case by case.

## Ethics statement

The study complies with current Italian Laws.

## Additional file

Additional file 1:
**Radiobiological Model.**

## Abbreviations

EBRT: External beam radiation therapy
hSRT: Hypofractionated stereotactic radiotherapy
LQ: Linear-quadratic
SF: Surviving fraction
BED: Biologically effective dose
NTD: Normalized total dose
LQ-L: Linear quadratic-linear
NTCP: Normal tissue complication probability
DVH: Dose-volume histogram
TCP: Tumor control probability
Dmax: Maximal doses
