# Supplementary material for: CyberKnife multisession stereotactic radiosurgery and hypofractionated stereotactic radiotherapy for perioptic meningiomas: intermediate-term results and radiobiological considerations
Source: Springerplus. 2015 Jan 30;4:37. doi: 10.1186/s40064-015-0804-2 (PMC4320239; doi:10.1186/s40064-015-0804-2)
Supplement: Additional file 1: — Radiobiological Model. [file 40064_2015_804_MOESM1_ESM.docx]

**Additional file 1**

The linear-quadratic (LQ) formulation **e^-(αD+βDexp2)^** is often used to model biological response to radiation. For instance, when applied to single fraction cell survival studies the surviving fraction (SF) is generally expressed as:

eq. 1 **SF = e^-(αD+βDexp2)^**

where D is the dose in Gy, α is the cell kill per Gy of the initial linear component (on a log-linear plot) and β the cell kill per Gy^2^ of the quadratic component of the survival curve.

Curves for the individual LQ components e^-αD^ and e^-βD^**^exp^**^2^intersect at the dose where the αD and βD^2^ components of cell killing are equal. This intersection happens to occur at a dose equal to the ratio of α to β, and is often referred to in the literature simply as α/β.

The biological effect (E) per fraction (n) of fractional dose (D) can be expressed as:

eq. 2 **E_n_ = (αD+βD^2^)**

The biologically effective dose (BED or E/α) is an approximate quantity by which different radiotherapy fractionation regimens may be compared. For instance, for an EBRT regimen employing *n* equal fractions of conventional size the BED will be:

eq. 3 **BED = E/α = nD (1 + D /α/β)**

where n = number of fractions, D = dose/fraction, and nD = total dose.

The LQ model is based upon the assumption that the biological response to radiation can be described by an equation with two principal components, one that is proportional to dose and another that is proportional to square of dose. To relate the biological effect of a course of radiation to the total dose of radiation administered with the standard fractionation scheme (2 Gy/ fraction), the concept of normalized total dose (NTD) has been used. Expression is derived from the linear-quadratic model:

eq. 4 **NTD= BED/(1+d / αβ )**

To establish the equivalence with the standard fractionation scheme (2 Gy/fraction), in the second equation, d would take the value 2 Gy.

We calculated a target BED to be used in modified fractionation schemes. To calculate this target BED we analyzed the clinical data, currently available in the literature, on the irradiation of meningiomas. Sufficient clinical evidence could be found on the treatment in a single fraction and using conventional fractionation (2Gy/day). We selected two different dose/fraction schemes that resulted in similar tumor control rate. Using the LQ model, it was possible to calculate the α/β of meningiomas and the BED necessary to achieve optimal tumor control when using modified fractionation schemes.

Actually, assuming that the BED_1_ of the first treatment is equal to the BED_2_ for a tissue of unknown α/β,

eq. 5 $\text{D1 (1+}\frac{d1}{\alpha/\beta}$)= $\text{D2 (1+}\frac{d2}{\alpha/\beta}$)

and

eq. 6 $\frac{\alpha}{\beta}=\frac{D1 \times d1-D2 \times d2}{D2-D1}$

Although BED calculations seems reliable for comparing conventional fractionation regimens, the standard LQ formulation upon which these calculations rests is suspect for fractions of very high dose. To better address hypofractionation regimens (e.g. SBRT, SRS) which employ very high dose fractions we used the linear quadratic-linear (LQ-L) model described by Astrahan ([Astrahan 2008](#_ENREF_3)) that describes the transition to a linear tail at high dose fractions.

To transition to a linear model for very high dose fractions the standard LQ model can be extended to a bipartite LQ_α/β_-L_DT_ form which can be expressed as:

**SF = e^-(αD+βDexp2)^** for fraction size D < D_T_

and  eq. 7 **SF = e^-[αD^_T_^+βD^_T_^exp2+γ(D−D^_T_^)]^** for fraction size D >= D_T_

eq. 8 **BED_n_ = [D + D^2^/(α/β)]** for fraction size D < D_T_

and  eq. 9 **BED_n_ = D_T_ + D_T_^2^/(α/β) + [(γ/α)(D−D_T_)]** for fraction size D >= D

where D_T_ is the dose at which the survival curve becomes linear and γ is the cell kill per Gy in this high dose linear portion of the survival curve. The term γ is thus reminiscent of D_0_ (the dose that reduces survival by 1/e in the final linear portion of the survival curve) of the older multitarget survival model, but is more in keeping with the Greek nomenclature of the LQ model. The γ/α ratio can be calculated from the tangent at D_T_ and the α/β term of the standard LQ model as:

eq. 10 **γ/α = 1 + [2D_T_/(α/β)]**

To calculate the tolerance of the optic nerve we used a similar approach. Basically, we reviewed the literature on the tolerance to a single fraction of radiations and to conventional fractionation. We therefore, selected two regimens with corresponding complication probability to extrapolate the α/β of the optic apparatus to interpolate clinical data to hypofractionated schemes.

Furthermore, we used the “optic ret” model proposed by Goldsmith, et al. ([Goldsmith et al. 1992](#_ENREF_13)). This is a model predicting the total dose associated with a “low risk” of optic neuropathy when various doses per fraction are used.

The optic ret is based on the total dose and the number of fractions used:

eq. 11 **optic ret dose = D (cGy)/N^0.53^**

where N is the number of fractions used and D is the total physical dose in cGy. Goldsmith et al.([Goldsmith et al. 1992](#_ENREF_13)), predicted, based on clinical data, that doses to the optic nerve or chiasm less than or equal to 890 optic ret would be safe. Therefore for a particular number of fractions, N, the optic tolerance would be predicted to be total dose D, where

eq. 12 **D = 890×N^0.53^**

This model predicts doses that are associated with a very low risk of optic neuropathy with single doses, 8.9 Gy, and up to at least 30 fractions, 54 Gy. This model is used to predict for optic nerve tolerance following various numbers of fractions in this analysis.

Normal tissue complication probability (NTCP) was calculated using the widely used NTCP model of Lyman. It is an empirical model employing four parameters and assumes that the probability of complications after uniform irradiation follows a sigmoid dose-response relationship; the formulation of the model is discussed in detail elsewhere ([Lyman 1985](#_ENREF_17); [Kutcher and Burman 1989](#_ENREF_15)).

To calculate an NTCP using the Lyman formalism, single dose/volume numbers are calculated as an intermediate step with a dose-volume histogram (DVH) reduction method. For example, the DVH representing nonuniform irradiation of the organ must be transformed to a “single-step” DVH with an effective volume (V_eff_) and a corresponding reference dose.

The resulting uniform single-step DVH gives an equivalent NTCP to the initial nonuniform DVH. This method is discussed by Kutcher et al. ([Kutcher and Burman 1989](#_ENREF_15)). Optic nerve, and optic chiasm tolerance values, summarized by Emami et al. ([Emami et al. 1991](#_ENREF_11)), gave tolerance doses for 5% (TD_5_) and 50% (TD_50_) chances of a complication happening in 5 years for whole organ irradiated to a uniform dose. Burman et al. ([Burman et al. 1991](#_ENREF_4)) fit the clinical data to yield parameters for use in the Lyman model and these were used in our calculations (optic nerve and chiasm: volume parameter n = 0.25, the fitting parameter m = 0.14, TD_50_ = 65 Gy).

The tumor control probability (TCP) calculation was based on the LQ model of the survival capacity of irradiated cells and on two assumptions: 1) the tumor control is achieved when there are not cell clones surviving after irradiation; 2) the cell death occurs randomly and the probability of surviving cells in the tumor follows the Poisson statistics.

Therefore, if **N(D) =N0× Sn**  is the number of cells surviving after irradiation the probability of tumor control can be expessed as:

eq. 13 **TCP=e^-KN^**

where K is a proportionality factor due to the possibility that there is a number of cells from which the tumor recurrence cannot occur because of environmental reasons. In terms of SF2, namely of cell survival after a daily dose of 2Gy, the survival after n fractions is

eq. 14 **S= (SF_2_)^n^= e^-nd(α+β×d)^**

Then

eq. 15 **TCP = EXP [-KN_0_ ^-nd(α+β×d)^]**
